# Recommended foods for male infertility in Iranian traditional medicine

**Published:** 2012-11

**Authors:** Fatemeh Nejatbakhsh, Esmaeil Nazem, Ashrafeddin Goushegir, Mohammad Mahdi Isfahani, Alireza Nikbakht Nasrabadi, Marzieh Baygom Siahpoosh

**Affiliations:** 1 *Department of Iranian Traditional Medicine, Tehran University of Medical Sciences, Tehran, Iran.*; 2 *Research Institute for Islamic and Complementary Medicine, Tehran University of Medical Sciences, Tehran, Iran.*; 3 *School of Nursing and Midwifery, Tehran University of Medical Sciences, Tehran, Iran.*

**Keywords:** *Iranian traditional medicine*, *Nutrition*, *Sexual dysfunction*, *Male infertility*

## Abstract

**Background: **Male infertility accounts for 30-50% of all infertilities among couples. Iranian traditional medicine (ITM) stressed the importance of nutrition in the prevention and treatment of male infertility. Many Iranian traditional physicians have described the traits of specific foods for prevention and treatment of male infertility.

**Objective:** To explore the principles and roles of foods recommended by ITM scientists in prevention and treatment of male infertility as well as enlisting all the recommended foods for treating this problem addressed through the ITM original resources written between 815 and 1901.

**Materials and Methods:** In this review study specific data related to the subject among all referral ITM texts was extracted firstly, and then the collected data were analyzed using inductive content analysis.

**Results:** The analysis of data revealed that foods that enhance sexual performance must have 3 properties; they should be warm in nature, very nutritious, and flatulent. Foods that are warm in nature and nutritious affect the quality and quantity of semen. A food having the third trait of being flatulent is required to complete sexual performance by creating an erection. Foods with only one of these traits must be consumed with another food that has the other trait. This study also provided a list of foods that can enhance the quality and increase the quantity of semen.

**Conclusion:** Foods that can enhance sexual performance and the quality and quantity of semen can be recommended to male patients who suffer from infertility in medical centers.

## Introduction

Infertility, defined as the inability to conceive a child after 1 year of having intercourse without using contraception, affects 10-15% of couples (1). Male infertility accounts for 30-50% of all infertilities and 30-40% of them are sperm disorders (1, 2).

Grand masters of ITM have categorized the causes of infertility into 6 groups. According to this categorization, one of the most important reasons of infertility is low or abnormal semen. According to Iranian traditional medicine (ITM) having low or abnormal semen has its own etiologies, which are hot intemperament, cold intemperament, moist intemperament, dry intemperament, heart weakness, stomach weakness, liver weakness, brain weakness, drug abuse, sexual inactivity, and psychosomatic disorders (3-12).

Although nutritional factors, such as food regimens known to affect fertility and nutrients recognized to affect molecular mechanisms and balance physiological functions, and have been documented the role of nutrients as treatment for male infertility, but there is no specific attention to nutritional recommendation for infertility among classical medicine references books (1, 13-15).

In contrast, ITM physicians have recommended various foods as treatment essentials for male infertility. Grand masters of ITM have always paid special attention to the role of nutrition in preventing and treating diseases. Abu Zeid Balkhi, who was Razi’s master, professed that food is the first thing to be noted and carefully considered in maintaining health. Under the section on “Treatment” he mentioned that drugs should be avoided unless it is absolutely needed, because unlike food, drugs are in contrast with human nature (16). Both Razi and Jorjani believed that patients must be treated with food as far as possible (17, 18).

The aim of this study was to explore the principles and roles of nutrition in prevention and management of male infertility according to the Iranian traditional medicine and also enlisting all the recommended foods along with their quality in treating this problem.

## Materials and methods

This study was review and an inductive content analysis which, sections of authentic and original books of ITM written between 815 and 1901 that focused on male infertility food regimens were selected according to the specific predetermined inclusion criteria (19-21).

The inclusion criteria were that the editors of the selected books, were known as prestigious experienced physicians of ITM with well practical experiences, and expressed subject eloquently. The selected books were assigned into 6 categories. The first group included 13 books about principles, diseases, and their treatments (6, 7, 9, 10, 12, 18, 22-28).The second group included 4 books that were only about principles (16, 29-31).The third group included 10 books about diseases and their treatments (3-5, 8, 11, 32-36).The fourth group included 8 books that especially considered sexual diseases and disorders (37-44). The fifth group included 5 books on vegetables, minerals, and animals (45-49). The sixth group included three books on specialized vocabulary of ITM (50-52).

There were extracted 1300 pages about the nutrition for prevention and treatment of this disorder in the selected reviewed books. After first stage reading of all the data, 140 pages of discussion were assembled, and then the extracted content was analyzed using inductive content analysis. Content analysis is a systematic technique for analyzing written texts to condense and organize the data (53). An inductive approach is recommended if there is insufficient knowledge about the subject or if the knowledge is fragmented. The key feature of this process is that many words of the text related to specific subject are classified into much smaller but more comprehensive content categories (54, 55).

The whole process of content analysis was started by thorough, line-by-line reading of the extracted materials, key sentences and concepts were defined and coded. In the next step, by constant comparison of data and codes, significant meaning units and primary themes were identified. The process of data analysis was repetitive, and main themes were identified after completely reading the content of the forms (by adding, removing, and merging primary data) and analyzing it. Peer checking was done to increase the rigor and credibility of the data. In addition, the authenticity of the interpretation and coding process was increased by consulting a research team. The content of the forms, codes, and themes were read, reviewed, and agreed upon by the consensus of the research team (56). The study was approved by Tehran University of Medical Sciences Ethical Committee.

## Discussion

ITM offers valuable information about nutrition and its role in preventing and treating male infertility. The analysis of results showed that nutrition has essential role in managing this problem. The results of this study are categorized into 4 groups.

- Preference of food therapy over drug therapy to increase the quality and quantity of semen in the treatment of male infertility. Nazem Jahan wrote, “For increasing sexual power, food has more advantages than drugs” (3). There are 4 reasons for this statement; the first is that only food can increase the quantity of semen because semen would be increased by material which is food, and any drug that can increase semen quantity is food-based. Avicenna also wrote, “Know that (for enhancing sexual power) food is the most trusted because it has more material and excitation power” (6). Liver performs some process on food and converts it to semen (6, 30, 57). Since semen is made from nutrients, drugs can only alter the semen’s nature; thus if it is intemperament, warmer, or colder than normal, then drugs can rectify it, but cannot help increase its quantity (39). Any drug that can increase semen quantity will have to have nutritional characteristics. Another reason is that drugs bring about changes in body quality, and can balance a hot or cold intemperament. However, drugs cannot maintain the balance of nature or strengthen it. This can be achieved by foods; but drugs that enhance the characteristic of sexual power alongside its quality, can do so too. The 3^rd^ reason is that while the amount of drugs is small, it is more powerful than the body’s nature, which is the managing power of the human body. The nature of the human body cannot benefit from something with these properties; it might even get in the way of nature, as is seen with the side effects of many drugs. Finally, food travels to the organs before other materials and is absorbed sooner than drugs are. Ali Ibn Rabban Tabari wrote, “Food gets to the body organs before other materials and gets absorbed sooner than drugs. We can use drugs that are similar to food” (12).
- Characteristics of foods that increase the quality and quantity of semen: Foods that increase the quality and quantity of semen would be placed in a subset of foods that improve sexual performance. Foods that improve sexual performance must have 3 properties; they must be warm in nature, flatulent, and very nutritious. Jorjani wrote, “Foods chosen for this purpose have 3 characteristics and these characteristics are necessary for them, the first one is to have lot of nutrition, the second one is to be flatulent, and the third one is to be warm-nature” (7). Foods with warm nature create heat in the body, warming the body nature. The food must be warm-natured because semen is warm-natured too. Avicenna wrote, “Men’s semen is hot, matured, and thick” (6). Therefore, only foods that have a similar nature can increase the semen quantity. Food’s warm nature must be balanced because extreme heat would dry the semen and prevent the creation of flatulence through vessels, which are needed in the creation of an erection. The food must be nutritious because the material must increase. Flatulence in the vessels is also considered as the reason for an erection, and this does not occur in the absence of flatulence. Avicenna and Nazem Jahan wrote, “The reason of erection is flatulence, which could occur by semen or something else, and cold-nature and hot-nature are both opposites of flatulence since cold-nature would prevent its generation and hot-nature would destroy it and only moderate moisture and enough heat can create flatulence” (37). Therefore, to increase the quality and quantity of semen, foods must be warm-natured and very nutritious, and being flatulent to help create erection is necessary to complete sexual function. Food is responsible for the body’s development and growth, and for making up the body’s losses in temperature and moisture. Therefore, food must have the temperature and moisture to make up for this loss. On the other hand, food gets to organs through the blood, which has a warm and moist nature (58). Since the semen has a warm nature, any food that is consumed to increase its quantity and maintain its nature must be warm in nature (6, 31). Semen must be warm for 3 reasons: for maintaining consistency, for coagulating, and for motility (6, 30, 57, 59). The second required characteristic of foods that increase the quality and quantity of semen is being nutritious. Foods that contribute to blood formation are more nutritious, because blood is responsible for feeding organs; therefore, more blood feeds organs better (45, 58). Since semen is made from food, the more available the food (nutritious), the more semen created (3).
- All 3 characteristics must exist to complete sexual function. Some foods have all 3 characteristics together, such as chickpeas, beans, turnips, and carrots. However, some foods have only 2 of the 3 characteristics; thus, they must be consumed with another food that has the third characteristic to complete their function. Broad beans are very nutritious and flatulent but cold natured; thus, they must be consumed with foods that are hot-natured, like cinnamon or onion, which is warm-natured and flatulent but not very nutritious; thus, it must be consumed with lamb. Nazem Jahan wrote, “Selected foods for solving this problem must have three properties; first is to be very nutritious; second, to be flatulent; and third, to be warm-natured. If there is a food with all of these characteristics it is the best, if not two or three different foods must be combined to reach the desired results” (3). Hence, being warm-natured and very nutritious is sufficient for foods that increase the quality and quantity of semen. However, it must be mentioned that for having a complete sexual performance, erection is necessary, and flatulence through the vessels of the penis makes erection happen (3, 6, 7). This flatulence is caused by foods in vessel digestion (6). Being flatulent is not always bad; it is naturally needed in the body. For example natural movements of the stool in the intestines need flatulence; however, this flatulence must be at a normal level, and if it increases, it becomes disturbing (30).
- Different kinds of foods that increase the quality and quantity of semen. There is a large group of foods that improve sexual performance named with different frequencies in the analyzed literature of ITM. Therefore, we categorized foods that improve sexual performance into 3 groups:

1. Foods that were mentioned and addressed in 10 or more original ITM references, such as chickpeas, beans, carrots, turnips, onions, hazelnuts, pistachios, almonds, walnuts, spruce seeds, sesame, chio nut (Pistacia atlantica Desf), broad beans, eggs, lamb, fish, milk, garden cress, figs, grapes, and coconut.
2. Foods that were mentioned in 5 to 9 ITM references, such as hen, shrimp, baby pigeons, partridge, duck, francolin, animal brain, wheat, honey, date, radish, leek, celery, mint, fenugreek, cardoon (Cynara Cardunculus L.), banana, safflower, and sugar.
3. Finally, foods that were mentioned just in less than 4 ITM references, like chicken, bone marrow, rice, basil, beet, kohlrabi (Brassieca Oleraca L.), molon (Cucumis Melo L.), mango, white mulberry (Morus Alba L.), pear, citron peel (Citrus Medica L. ), opium poppy, fresh cheese, garlic (for moist natures), grass pea vin (Lathyrus Sativus L.), egyptian colcocasie (Colcocasia Esculenta (L.) Schott.), and flixweed seed (Descureania Sophia (L.) Schur.).

The foods named as semen-increasers were chickpeas, beans, carrots, turnips, onions (fried in oil), pistachios, almonds, spruce seeds, sesame, shrimp, fish, hen, baby pigeon, partridge, duck, francolin, animal brain, rice, milk, radish, leek, garden cress, cardoon (Cynara Cardunculus L.), kohlrabi (Brassieca Oleraca L.), grape, banana, coconut, safflower (its juice mixed with fig juice), garlic (for moist natures), and egyptian colcocasie (Colcocasia Esculenta (L.) Schott.) (5, 8, 10, 16, 22-28, 30-49).

However, this does not mean that other foods that improve sexual performance would not increase semen. For example, eggs, wheat, dates, and honey are very nutritious and warm-natured; thus, they can generate and increase semen but they are not identified in these groups (45). Some foods have only one or two needed characteristics, and they must be combined with other foods to complete their action (3). 

## Conclusions

The consensus of ITM is that foods that are warm-natured, flatulent, and very nutritious can be used to prevent or treat male infertility. Hence, it is recommended that men use these introduced foods to prevent infertility and those men who are infertile, start consuming these foods to improve their condition. It is recommended that healthcare team provide a list of these foods based on their local habitat and the availability of these foods for infertile patients
